# Improving the Reproducibility of the Standing Forward Flexion Test After Specific Training: A Cross-Sectional Study

**DOI:** 10.7759/cureus.99437

**Published:** 2025-12-17

**Authors:** Saverio Colonna, Antonio D'Alessandro, Francesca Pozzati, Michela Venturi, Veronica Andrenacci, Doris Romeo

**Affiliations:** 1 Rehabilitation Medicine, Spine Center, Bologna, ITA; 2 Research and Development, Osteopathic Spine Center Education (OSCE), Bologna, ITA; 3 Anatomical Sciences, Osteopathic Spine Center Education (OSCE), Bologna, ITA

**Keywords:** functional evaluation, inter-examiner agreement, intra-examiner reproducibility, manual therapy, osteopathic assessment, palpation reliability, palpatory training program, posterior superior iliac spine (psis), sacroiliac joint dysfunction, standing forward flexion test

## Abstract

The standing forward flexion test (SFT) is commonly used in osteopathic and manual therapy practice to assess sacroiliac joint mobility, yet its reliability has long been questioned due to inconsistent inter- and intra-examiner agreement. This cross-sectional study evaluated whether a structured palpatory training program could improve the reproducibility of the SFT and analyzed which phase of the test-trunk flexion or standing contributes most to variability. Two senior osteopathy students independently assessed the posterior superior iliac spines (PSIS) of 72 asymptomatic participants across three sessions: baseline (T0), after 25 hours of specific training (T1), and final assessment (T2). In addition to the classical SFT, separate measurements were performed for each phase, allowing phase-specific comparison and the development of a “reverse SFT” procedure, in which palpation is executed first in flexion and then in standing. Agreement was quantified using weighted Cohen’s κ, Gwet’s AC2, and Brennan-Prediger κ. At baseline, reliability was poor (κw ≈ 0.14), consistent with previous literature. After training, both intra- and inter-examiner reproducibility increased substantially (κw = 0.60-0.83; AC2 = 0.60-0.82; Po = 0.81-0.92), with slightly higher agreement in flexion than in standing. These results demonstrate that palpatory variability is not an intrinsic limitation of manual assessment but reflects insufficient training and methodological inconsistency. A structured 25-hour consensus-based program can markedly improve diagnostic reproducibility, and the reverse SFT may represent a more stable and standardized approach for both educational and clinical use.

## Introduction

The topic of diagnostic accuracy in clinical practice has been described as a “new frontier” by the British Medical Journal [1], highlighting the growing focus on the validity and reproducibility of assessment procedures. Osteopathy, and manual therapy more broadly, cannot exempt itself from these principles. Kmita et al. [2] emphasized that the reliability of palpatory techniques represents a crucial issue for the scientific credibility and clinical legitimacy of practice in this field.

For clinical tests to have true diagnostic value, it is essential that they are reliable [3]. Reliability refers to the consistency with which a measurement is performed [4]. Indeed, a diagnostic procedure is defined as highly reliable when it produces consistent results under similar conditions. However, it should be noted that reproducibility alone does not guarantee the validity of a test. Reproducibility and validity are complementary properties that determine the methodological robustness of a clinical test. Reproducibility refers to the extent to which repeated measurements yield consistent results under comparable conditions, encompassing both intra- and inter-examiner agreement and serving as a prerequisite for any reliable clinical assessment [5,6]. Validity, in contrast, concerns the degree to which a test measures what it is intended to measure and therefore depends on the accuracy with which the clinical phenomenon of interest is captured [7,8]. A test may be reproducible without being valid, but it cannot be valid without first demonstrating an acceptable level of reproducibility - an aspect that remains central to the evaluation of manual examination procedures.

Standing forward flexion test (SFT): literature review

Among the various methods used to assess pelvic mobility, the SFT and the seated flexion test (TFS) are the most widely employed and recognized tools in the osteopathic literature [9-17]. Under physiological conditions, the sacroiliac joint (SIJ) allows sacral flexion while maintaining the symmetry of the posterior superior iliac spines (PSIS). During anterior pelvic tilt, the sacrum flexes forward while the PSIS remain relatively stable. However, dysfunction of the SIJ may alter this dynamic, producing an anterior dragging of the iliac component and a greater cranial excursion of the PSIS on the involved side [12,18].

PSIS: reliability of palpation

Given the central role attributed to PSIS palpation, Cooperstein et al. [5] conducted a systematic review examining intra- and inter-examiner reliability for PSIS localization and side-to-side asymmetry assessment. The review included studies performed in seated [19-21], prone [2,22-26], standing [27-29], and combined positions [18]. Across these investigations, PSIS assessment methods varied substantially, including bilateral symmetry judgments, single-point localization, and linear distance measurements, often without consistent application of robust statistical techniques [18-29].

Importantly, none of the included studies achieved inter-examiner reliability levels considered “substantial” according to the Landis and Koch scale [30]. In studies using kappa statistics, the sample size-weighted mean kappa was low (κ=0.27), with intra-examiner reliability consistently exceeding inter-examiner reliability [5]. A non-significant trend suggested that higher methodological quality might be associated with slightly better agreement (r=0.43), although clinically meaningful reliability thresholds were never reached [30].

These findings are consistent with other systematic reviews on spinal landmark palpation [31] and with evidence showing that examiner experience, academic background, and shared methodology do not substantially improve reliability [2,28,32]. Training-based interventions have yielded mixed results. While brief training programs appear insufficient to meaningfully enhance agreement [22,33], longer consensus-based protocols have demonstrated moderate improvements for selected palpatory tests, though reliability for positional asymmetry remains limited [34].

In light of this evidence, the primary objective of the present study was to determine whether a specific and standardized palpatory training program could improve intra- and inter-examiner agreement in the localization of the PSIS under commonly used clinical conditions, specifically with the subject in the standing position as assessed during the SFT.

## Materials and methods

Two students in their fifth year of study at the Osteopathic Spine Center Education (OSCE) school in Bologna, Italy, participated as examiners, while three additional students assisted with data collection. The number of subjects recruited was set at a minimum of 40 per assessment session, in accordance with the guidelines of the International Academy of Manual/Musculoskeletal Medicine, which recommends a sample size sufficient to ensure adequate statistical power for palpatory reliability studies [33].

Inclusion criteria consisted of subjects aged between 18 and 40 years, in good general health, free from lumbopelvic pain or previous musculoskeletal disorders, and available to participate in all experimental phases of the study.

Exclusion criteria included a clinical history of significant lumbar or pelvic trauma, as well as neurological or musculoskeletal conditions that could interfere with the evaluation.

The sample was divided according to the assessment phases: 40 subjects participated in the baseline evaluation, 20 subjects took part in the intermediate verification phase, and 48 subjects in the final evaluation. Some subjects participated in only one assessment session, others in two, and a few in all three sessions. In total, 72 different individuals were included in the study.

Although the study was originally conceived as cross-sectional, the repeated assessments performed before and after a structured training program make it more accurately described as a prospective repeated-measures observational design aimed at evaluating the effect of examiner training on test reproducibility.

Project phases

**Figure 1:**
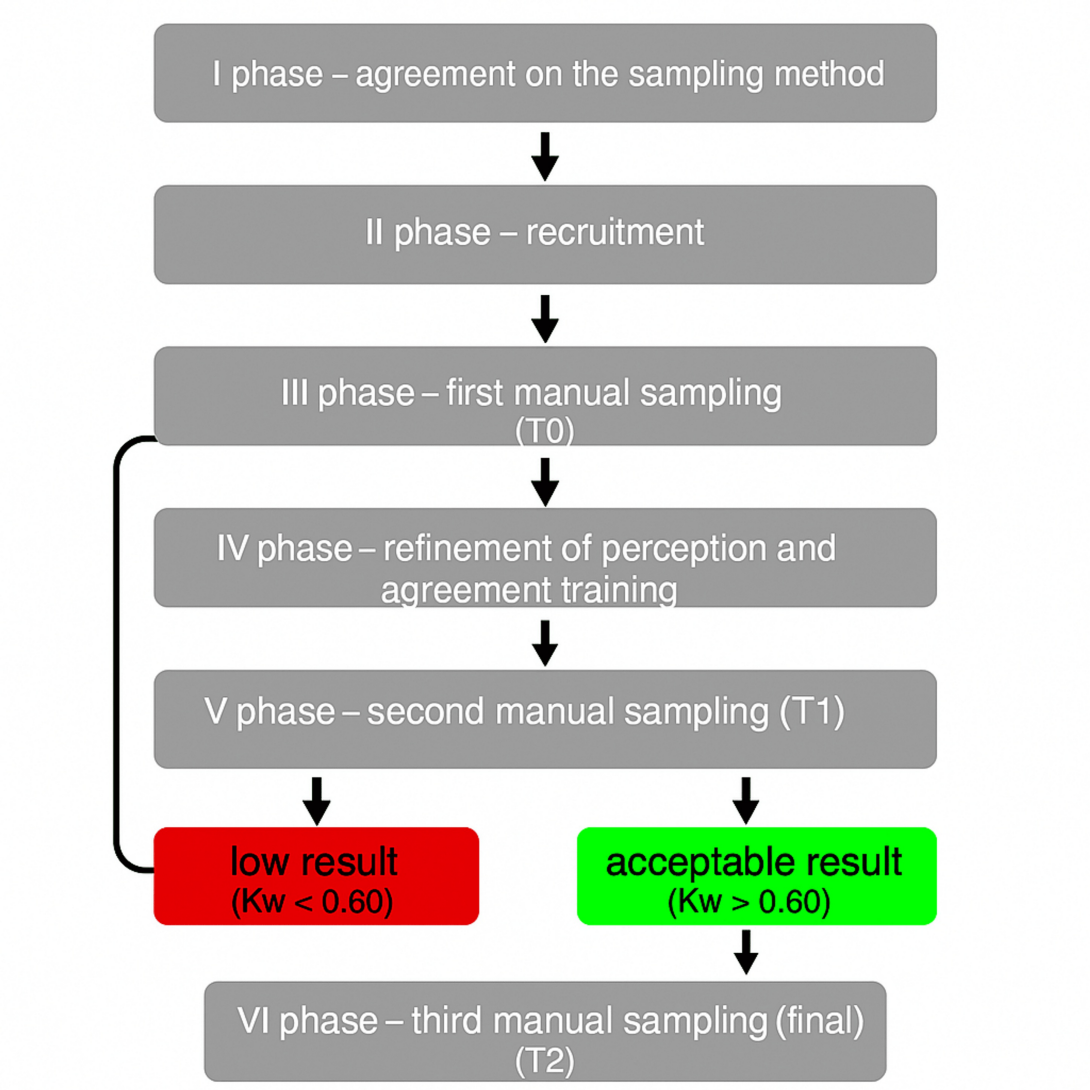
Flow diagram of the experimental protocol. The diagram illustrates the sequential phases of the study. Image credit: Saverio Colonna

The research project was structured into six sequential phases, each with specific objectives, procedures, and tools. The flowchart supporting the experimental protocol is shown in Figure 1.

The gradual planning of the phases aimed to ensure methodological consistency, reliability of measurements, and statistical validity of the collected data.

Phase I - Definition and Standardization of the Sampling Protocol

In the first phase, the examiners, together with the instructor of Palpatory Anatomy, participated in a two-hour training session designed to standardize the criteria for locating anatomical landmarks (particularly the PSIS) and the amount of digital pressure to be applied during palpation.

In the literature, the SFT has been described as the evaluation of PSIS movement from the neutral standing position (first assessment) to the end of trunk flexion (second assessment) [14,18]. Following the recommendations of several classical authors [35,36], the lower aspect of the PSIS was selected as the palpation target. Specifically, the test is considered positive on the side where the PSIS, when comparing the flexed to the extended position, moves more cranially and ventrally.

In this phase, the execution method of the SFT - the key element of the present study - was also standardized.

Phase II - Participant Recruitment

Participant recruitment was carried out at the OSCE School of Osteopathy in Bologna. Volunteers were identified through in-class announcements and communications disseminated via the school’s social media channels, including the Facebook group “Pianeta OSCE” and WhatsApp chats organized by academic year. This multi-level recruitment strategy was adopted to maximize the study’s visibility and ensure adequate participation.

Phase III - Baseline Assessment (T0)

The first assessment was conducted on a sample of 40 subjects, in line with the methodological recommendations of Patijn [33]. However, of the 40 subjects initially enrolled in the study, four participants dropped out due to absence at the second measurement session, reducing the final sample size of the first assessment to 36 subjects. Of these 36 subjects, 29 were male (80.6%) and seven were female (19.4%), with a mean age of 26.9 ± 5.7 years, a mean weight of 74.7 ± 11.7 kg, a mean height of 176.5 ± 7.6 cm, and a mean BMI of 23.5.

**Figure 2:**
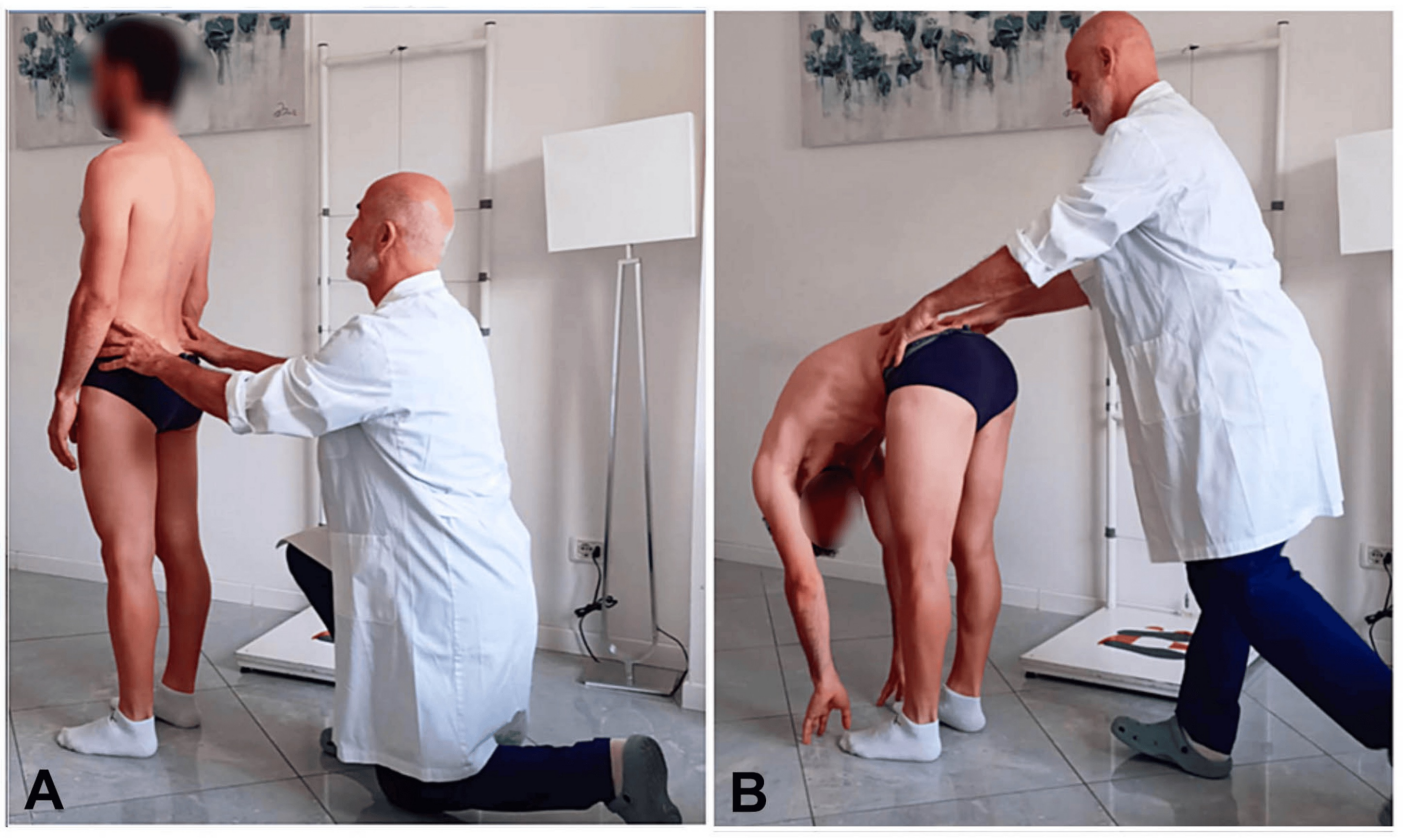
Example of the data collection procedure during the standing forward flexion test (SFT). A) Assessment of the posterior superior iliac spine (PSIS) level in the standing position. B) Assessment of the PSIS level in the flexed position. Image credit: Saverio Colonna

Description of the sampling procedure: Participants underwent a baseline assessment (T0), which consisted of the palpatory evaluation of the position of the PSIS in both the starting standing position and at the end of trunk flexion, performed by the two examiners. Given the poor repeatability of this test reported in the literature, we decided to employ two different measurement approaches: 1) Classical method: the examiners defined the result based on the presence or absence of asymmetric PSIS movement, classifying the outcome as negative when both PSIS moved symmetrically (positive right when the right PSIS moved more cranially and ventrally than the left, and positive left when the left PSIS moved more cranially and ventrally than the right); and 2) Dual assessment: in this version, the PSIS level was recorded first in the standing position and then at the end of maximal trunk flexion (Figure 2).

This approach was adopted to determine which assessment phase contributed most to the lack of agreement between examiners.

Measurements were classified on a five-level ordinal scale: +2: greater cranial-ventral height on the right side, with a difference of more than 10 mm compared to the left; +1: greater cranial-ventral height on the right side, with a difference between 5 mm and 10 mm compared to the left; 0: difference less than 5 mm; -1: greater cranial-ventral height on the left side, with a difference between 5 mm and 10 mm compared to the right; and -2: greater cranial-ventral height on the left side, with a difference of more than 10 mm compared to the right. These categories were established during the palpatory consensus phase and correspond to 5 mm increments between PSIS levels in terms of cranial-ventral displacement.

**Figure 3:**
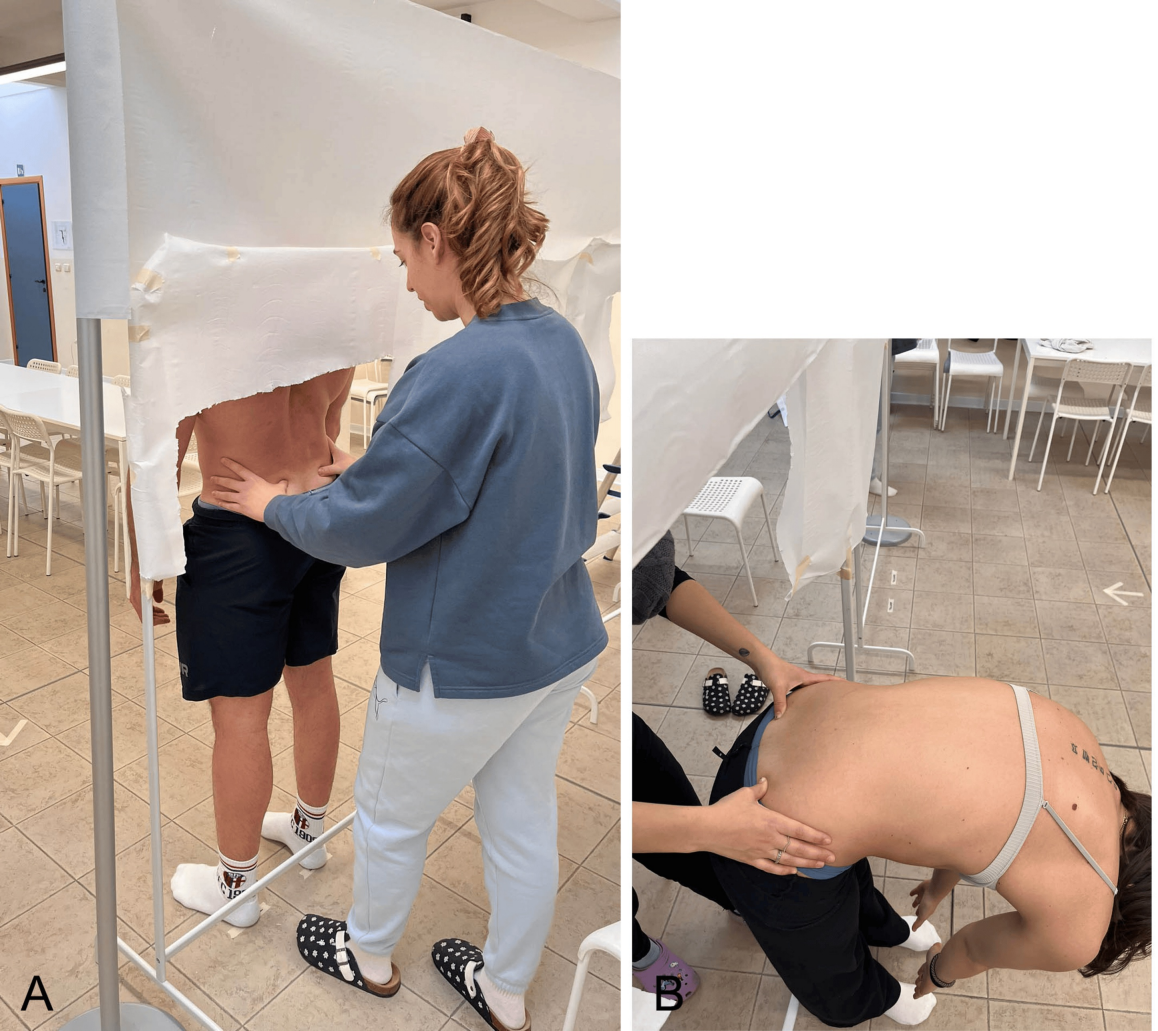
Sampling setup. The image highlights the sampling method designed to reduce subject recognition and limit bias for intra-examiner repeatability: A) postero-lateral view and B) anterior view. Image credit: Saverio Colonna

During T0, two separate evaluations were performed on the same subjects, at least two hours apart. When participants entered the examination room, the examiners faced away to prevent recognizing the subject. All measurements were conducted in a room with privacy screens, exposing only the pelvic region and lower limbs while the subject stood upright (Figure 3).

Phase IV - Training and Consensus Consolidation

**Figure 4:**
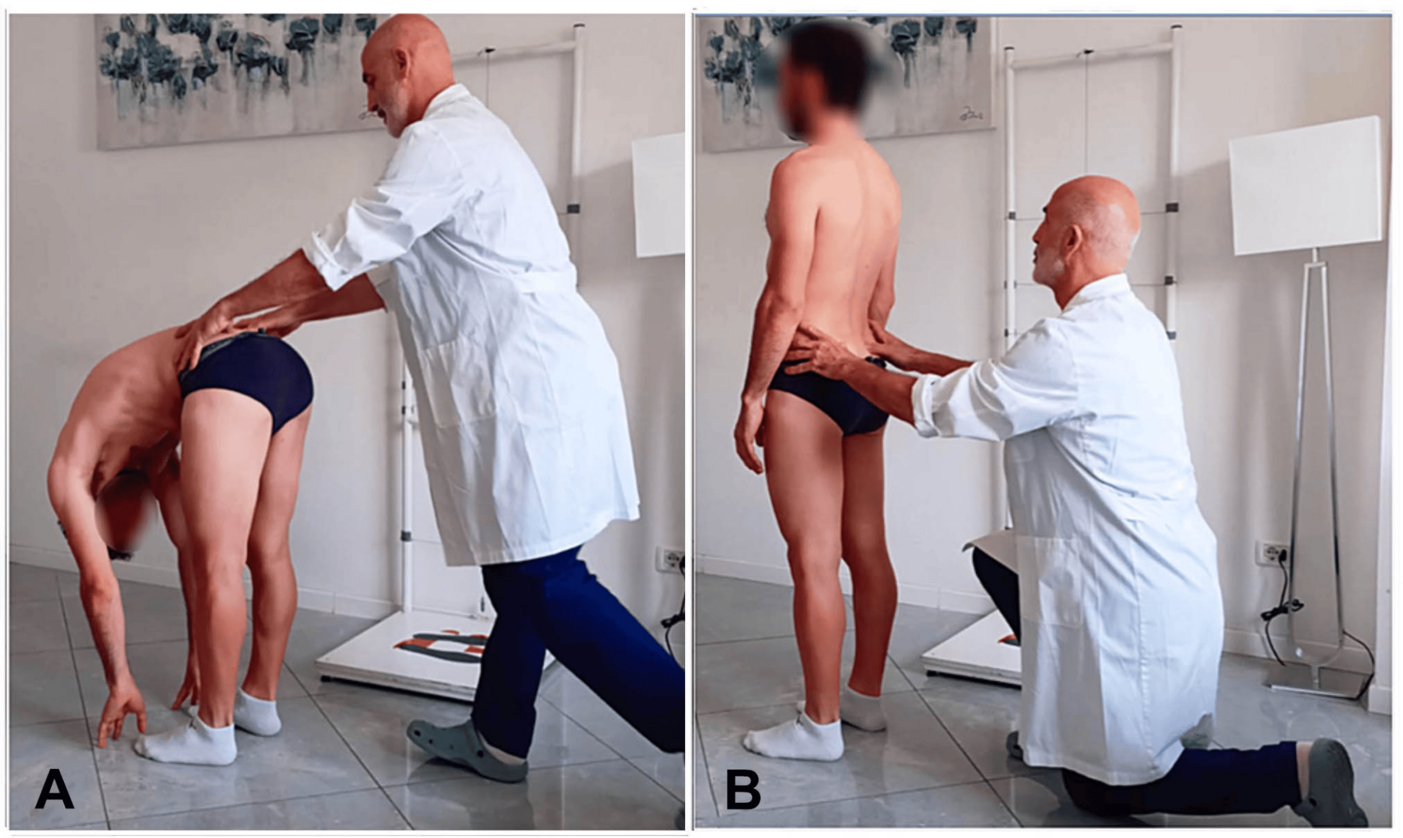
Reverse standing forward flexion test (SFT). Example of the posterior superior iliac spine (PSIS) palpation procedure illustration showing the palpation of the PSIS: first in flexion (A), followed by standing (B). Image credit: Saverio Colonna

In the fourth phase, the examiners participated in a 25-hour training program under the supervision of the scientific coordinator. The training included palpatory sensitivity exercises, visual recognition of asymmetries using laser devices, and practical applications on anatomical models and real subjects, as previously suggested in our earlier study [37]. As proposed by other authors [38], active involvement of the examiners in discussing how to perform the data collection sequence was encouraged to develop a procedure tailored to their needs. This collaborative approach proved advantageous in improving perception and, consequently, agreement. In particular, it led to the decision to reverse the order of data collection, starting with the flexed position, followed by the standing position. This modified procedure was defined as the reverse SFT (Figure 4).

For each exercise, the operators had the opportunity to experiment with different strategies, both in terms of palpation (using palms, fingertips, with elbows supported or suspended, etc.) and visual approach (with eyes closed, both eyes open, or only the dominant eye). Additional exercises were designed to refine the spatial perception of the palpated object, integrating tactile and visual components to enhance spatial awareness and both intra- and inter-examiner palpatory agreement.

Phase V - Intermediate Verification Assessment (T1)

The second assessment, defined as the intermediate phase, was conducted on a smaller sample of approximately 20 subjects, as proposed in the literature [33]. The procedure replicated that, of the first assessment, with the possibility of including either previously evaluated participants or new volunteers. The main objective of this phase was to verify the progression of manual agreement between examiners. If Cohen’s weighted kappa coefficient exceeded 0.6, the protocol advanced to the final assessment phase. Otherwise, the training program was to be extended by an additional four hours.

Phase VI - Final Assessment (T2)

The third and final assessment involved 48 subjects. As in the initial assessment, anthropometric parameters (age, weight, height, and BMI) were recorded at the beginning of the session. Of the 48 participants (30 males and 18 females), the mean age was 25.8 ± 5.4 years, with a mean weight of 72.1 ± 8.1 kg, a mean height of 1.70 ± 0.1 m, and a mean BMI of 23.5.

During the experimental procedure, the examiners were blinded both to their own previous evaluations and to those of the other examiner, thereby minimizing observer and recall bias. The total duration required to complete all six phases of the study was approximately six months.

Statistical analysis

The collected data were first subjected to a descriptive analysis to characterize the sample in terms of age, body weight, height, and BMI. Raw data were tabulated and organized into contingency tables using Microsoft Excel® (Microsoft® Corp., Redmond, WA). The study was designed and reported in accordance with the Guidelines for Reporting Reliability and Agreement Studies (GRRAS) [3].

The test results were reported in two ways: first, according to the classical description found in osteopathic literature, which compares the relative position of the PSIS between the standing and flexed postures (0=negative: at the end of trunk flexion, the difference between the two PSIS was less than 5 mm; +1 = positive right: the right PSIS moved more cranially and ventrally than the left; −1 = positive left: the left PSIS moved more cranially and ventrally than the right); and second, through a separate evaluation of the standing and flexed positions, each categorized into five levels as described in the data collection procedures.

Given the low repeatability of the SFT reported in the literature, and considering that the final test outcome depends on two separate measurements (standing and flexion), we assessed intra- and inter-examiner repeatability for each phase individually. With this approach, we aimed to determine which of the two measurement phases might have the greatest influence in undermining agreement on the final test outcome.

Given the ordinal nature of the measurement scale (ranging from - 1 to + 1 or - 2 to + 2, where 0 represented pelvic symmetry), Cohen’s weighted kappa (κw) with quadratic weights was used to quantify agreement. This weighting scheme accounts for the magnitude of disagreement between raters and provides a correlation-like interpretation suitable for ordinal data [39].

For each pairwise comparison (intra- and inter-examiner), the percentage agreement (Po) was calculated as a descriptive measure, followed by κw as the primary reliability coefficient. The Gwet’s AC2 coefficient, also computed with quadratic weighting, was included to provide a more stable reliability estimate less sensitive to the “prevalence and bias paradox” that can distort κ values [40]. This approach has been empirically validated in studies showing that AC2 maintains higher consistency under skewed marginal distributions [41]. Both Cohen’s weighted κ and Gwet’s AC2 were calculated using quadratic weights for the five-level (-2 to +2) and three-level (-1, 0, +1) ordinal scales, as partial disagreement (e.g., -1 vs 0) was considered less relevant than maximal disagreement (-1 vs +1), in order to preserve the ordinal nature of the data and ensure comparability between coefficients.

To further mitigate the prevalence and bias effects, the Brennan-Prediger coefficient (BP-κ) was calculated as a generalized version of the Prevalence-Adjusted Bias-Adjusted Kappa (PABAK) for multiple categories [42,43]. For interpretive comparison, a binary version of the data (symmetrical vs. asymmetrical) was also analyzed to obtain PABAK values directly comparable to those used in clinical palpation studies [44].

Prevalence Index (PI) and Bias Index (BI) were calculated following the method of Byrt et al. [43] to quantify category imbalance and examiner tendency, respectively.

$$ PI = \left| \frac{(n_{11} + n_{10}) - (n_{01} + n_{00})}{N} \right| $$

$$ BI = \left| \frac{(n_{11} + n_{01}) - (n_{10} + n_{00})}{N} \right| $$

Here, n_11_ denotes the number of observations in which both examiners scored the finding as present; n_00​ _those in which both scored it as absent; n_10_​ cases in which examiner A scored “present” and examiner B “absent”; n_01​_ the reverse pattern; and N the total number of observations.

For the prevalence and bias indices, the five-/three-level ordinal ratings were dichotomized as Neutral (0) vs Non-neutral (±1, ±2). We report the Prevalence Index (PI = |a−d|/N) and Bias Index (BI = |b−c|/N) from the resulting 2×2 tables, where a and d are agreement cells and b and c are disagreement cells.

All coefficients were interpreted according to Landis and Koch [30], with thresholds defining poor (<0), slight (0.00-0.20), fair (0.21-0.40), moderate (0.41-0.60), substantial (0.61-0.80), and almost perfect (0.81-1.00) agreement. The Landis-Koch scale was used and appropriately cited; no permissions were required.

Post-hoc precision/power. Given n=48 (T2) and n=36 (T0), for five- and three-level ordinal ratings with reasonably balanced category distributions and expected agreement around κ≈0.6, the 95% CI width is expected at ≈0.18-0.24 (T2) and ≈0.22-0.30 (T0). This corresponds to ~80% (T2) and ~70-75% (T0) power to distinguish κ=0.6 from κ=0.4, which we considered adequate for reliability reporting.

Statistical computations and reliability indices were performed using Python (v3.11) with custom routines based on standard formulas for κw, AC2, and BP-κ.

## Results

Tables 1-2 report the intra- and inter- examiner agreement values for the classical assessment of the SFT, based on the detection of the PSIS that moves more cranially and ventrally.

**Table 1:** Intra- and inter-examiner agreement values for the two assessments per examiner in 36 subjects at time T0, evaluated according to the classical model. Po, observed agreement; Po SE, standard error of observed agreement; Po 95% CI (L, U), lower and upper limits of the 95% confidence interval for Po (Wilson method); Cohen’s κ (weighted), weighted Cohen’s kappa coefficient; κ SE, standard error of κ; κ 95% CI (L, U), lower and upper limits of the 95% confidence interval for κ (bootstrap method); Gwet’s AC2 (weighted), weighted Gwet’s Agreement Coefficient 2; AC2 SE, standard error of AC2; AC2 95% CI (L, U), lower and upper limits of the 95% confidence interval for AC2 (bootstrap method); BP-κ (k=3), Byrt–Pandit kappa for a 3-level scale adjusted for prevalence and bias; BP-κ SE, standard error of BP-κ; BP-κ 95% CI (L, U), lower and upper limits of the 95% confidence interval for BP-κ; PI: Prevalence Index; BI: Bias Index (both computed on binary data: symmetrical (0) vs asymmetrical (≠0)

Comparison	N	Po	Po SE	Po 95% CI (L)	Po 95% CI (U)	Cohen’s κ (weighted)	κ SE	κ 95% CI (L)	κ 95% CI (U)	Gwet’s AC2 (weighted)	AC2 SE	AC2 95% CI (L)	AC2 95% CI (U)	BP-κ (k=3)	BP-κ SE	BP-κ 95% CI (L)	BP-κ 95% CI (U)	PI (0 vs ≠0)	BI (0 vs ≠0)
Intra — Classic TFE (Examiner 1, I vs II)	36	0.52	0.08	0.37	0.68	0.14	0.13	-0.11	0.41	0.14	0.13	-0.12	0.40	0.29	0.12	0.05	0.52	0.22	0
Intra — Classic TFE (Examiner 2, I vs II)	36	0.33	0.07	0.202	0.49	-0.18	0.09	-0.37	-0.00	-0.19	0.09	-0.41	-0.02	0	0.11	-0.19	0.24	0.16	0
Inter — Classic TFE (Examiner 1 vs 2, I)	36	0.41	0.08	0.271	0.57	-0.03	0.11	-0.26	0.18	-0.03	0.11	-0.29	0.16	0.12	0.12	-0.09	0.36	0.19	0.02
Inter — Classic TFE (Examiner 1 vs 2, II)	36	0.44	0.08	0.295	0.60	0.00	0.13	-0.28	0.23	-0.00	0.14	-0.31	0.22	0.16	0.12	-0.05	0.40	0.19	0.02

**Table 2:** Intra- and inter-examiner agreement values from two assessments per examiner in 48 subjects performed at time T2 (final session), evaluated according to the classical model. Abbreviations are as in Table 1.

Comparison	N	Po	Po SE	Po 95% CI (L)	Po 95% CI (U)	Weighted κ (quadratic)	κ SE	κ 95% CI (L)	κ 95% CI (U)	Gwet’s AC2 (quadratic)	AC2 SE	AC2 95% CI (L)	AC2 95% CI (U)	BP-κ (k=3)	BP-κ SE	BP-κ 95% CI (L)	BP-κ 95% CI (U)	PI (0 vs ≠0)	BI (0 vs ≠0)
Intra — Classic TFE (Examiner 1, I vs II)	48.00	0.81	0.06	0.68	0.90	0.60	0.12	0.32	0.81	0.60	0.13	0.31	0.81	0.72	0.09	0.52	0.85	0.40	0.02
Intra — Classic TFE (Examiner 2, I vs II)	48.00	0.92	0.04	0.80	0.97	0.82	0.09	0.62	0.96	0.82	0.09	0.61	0.96	0.88	0.06	0.71	0.95	0.40	0.02
Inter — Classic TFE (Examiner 1 vs 2, I)	48.00	0.88	0.05	0.75	0.94	0.73	0.11	0.49	0.91	0.73	0.11	0.48	0.91	0.81	0.07	0.63	0.91	0.42	0.00
Inter — Classic TFE (Examiner 1 vs 2, II)	48.00	0.83	0.05	0.70	0.91	0.65	0.12	0.40	0.86	0.65	0.12	0.39	0.86	0.75	0.08	0.56	0.87	0.38	0.00

Table 3 (three categories) and Table 4 (five categories) report the agreement values for the baseline evaluation (T0) of PSIS height measurements obtained separately - first in trunk flexion and second in the standing position (reverse SFT) - by the two examiners in a sample of 36 subjects.

**Table 3:** Intra- and Inter-examiner reliability coefficients for the SFT at T0 (basal assessment), using the three-category scale. Abbreviations as in Table 1.

Comparison 3-category scale	N	Po	Po SE	Po 95% CI (L)	Po 95% CI (U)	Weighted κ (quadratic)	κ SE	κ 95% CI (L)	κ 95% CI (U)	Gwet’s AC2 (quadratic)	AC2 SE	AC2 95% CI (L)	AC2 95% CI (U)	BP-κ (k=3)	BP-κ SE	BP-κ 95% CI (L)	BP-κ 95% CI (U)	PI (0 vs ≠0)	BI (0 vs ≠0)
Intra Examiner 1 — Flex (I vs II assess)	36	0.472	0.083	0.32	0.63	0.138	0.146	-0.16	0.404	0.117	0.151	-0.198	0.363	0.208	0.125	-0.02	0.445	0.194	0.028
Intra Examiner 1 — Stand (I vs II assess)	36	0.472	0.083	0.32	0.63	-0.09	0.164	-0.418	0.223	-0.095	0.167	-0.443	0.215	0.208	0.125	-0.02	0.445	0.194	0.194
Intra Examiner 2 — Flex (I vs II assess)	36	0.389	0.081	0.248	0.551	-0.027	0.179	-0.391	0.31	-0.043	0.187	-0.417	0.303	0.083	0.122	-0.128	0.327	0.056	0.167
Intra Examiner 2 — Stand (I vs II assess)	36	0.611	0.081	0.449	0.752	-0.371	0.152	-0.625	0	-0.381	0.145	-0.643	-0.066	0.417	0.122	0.173	0.628	0.528	0.194
Inter — Flex I assess (Examiner 1 vs Examiner 2)	36	0.361	0.08	0.225	0.524	0.059	0.15	-0.239	0.341	0.057	0.156	-0.265	0.341	0.042	0.12	-0.163	0.286	0.056	0.167
Inter — Stand I assess (Examiner 1 vs Examiner 2)	36	0.528	0.083	0.37	0.68	0.146	0.179	-0.216	0.5	0.132	0.192	-0.254	0.497	0.292	0.125	0.055	0.52	0.167	0.167
Inter — Flex II assess (Examiner 1 vs Examiner 2)	36	0.528	0.083	0.37	0.68	0.343	0.132	0.024	0.556	0.343	0.135	0.022	0.553	0.292	0.125	0.055	0.52	0.194	0.028
Inter — Stand II assess (Examiner 1 vs Examiner 2)	36	0.611	0.081	0.449	0.752	0.14	0.098	-0.027	0.333	0.113	0.104	-0.068	0.316	0.417	0.122	0.173	0.628	0.556	0.167

**Table 4:** Intra and Inter-examiner reliability coefficients for the SFT at T0 (basal assessment), using the 5-category scale. Abbreviations as in Table 1.

Comparison 5-category scale	N	Po	Po SE	Po 95% CI (L)	Po 95% CI (U)	Weighted κ (quadratic)	κ SE	κ 95% CI (L)	κ 95% CI (U)	Gwet’s AC2 (quadratic)	AC2 SE	AC2 95% CI (L)	AC2 95% CI (U)	BP-κ (k=5)	BP-κ SE	BP-κ 95% CI (L)	BP-κ 95% CI (U)	PI (0 vs ≠0)	BI (0 vs ≠0)
Intra Examiner 1 — Flex (I vs II)	36	0.472	0.083	0.32	0.63	0.051	0.181	-0.3	0.379	0.036	0.177	-0.307	0.342	0.34	0.104	0.15	0.537	0.194	0.028
Intra Examiner 1 — Stand (I vs II)	36	0.472	0.083	0.32	0.63	-0.155	0.174	-0.491	0.206	-0.157	0.174	-0.509	0.204	0.34	0.104	0.15	0.537	0.194	0.194
Intra Examiner 2 — Flex (I vs II)	36	0.389	0.081	0.248	0.551	0.01	0.146	-0.293	0.264	-0.016	0.151	-0.347	0.24	0.236	0.102	0.06	0.439	0.056	0.167
Intra Examiner 2 — Stand (I vs II)	36	0.611	0.081	0.449	0.752	-0.371	0.152	-0.625	0.00	-0.381	0.145	-0.643	-0.066	0.514	0.102	0.311	0.69	0.528	0.194
Inter — Flex (Examiner 1 vs Examiner 2, I)	36	0.333	0.079	0.202	0.497	0.045	0.151	-0.238	0.335	0.043	0.155	-0.254	0.335	0.167	0.098	0.003	0.371	0.056	0.67
Inter — Stand (Examiner 1 vs Examiner 2, I)	36	0.528	0.083	0.37	0.68	0.139	0.163	-0.211	0.477	0.121	0.176	-0.236	0.463	0.41	0.104	0.213	0.6	0.167	0.167
Inter — Flex (Examiner 1 vs Examiner 2, II)	36	0.528	0.083	0.37	0.68	0.343	0.132	0.024	0.556	0.343	0.135	0.022	0.553	0.41	0.104	0.213	0.6	0.194	0.028
Inter — Stand (Examiner 1 vs Examiner 2, II)	36	0.611	0.081	0.449	0.752	0.14	0.098	-0.027	0.333	0.113	0.104	-0.068	0.316	0.514	0.102	0.311	0.69	0.556	0.167

Following the 25-hour training program, an intermediate verification assessment (T1) was conducted on a smaller sample of participants (20 subjects) to evaluate the progression of manual agreement between examiners. The procedure replicated that of the baseline assessment (T0) and served as a methodological checkpoint before proceeding to the final phase.

The results of the second data collection (T1) ranged from the lowest value (Kw = 0.88), obtained in the inter-examiner assessment with the three-level scale in the standing position during the second measurement by Examiner 1, to the highest value (Kw = 0.95), also in the inter-examiner assessment with the three-level scale during the second measurement by the same examiner, but this time in the flexed position. The results confirmed that both intra- and inter-examiner agreement achieved weighted Cohen’s kappa values exceeding the predetermined threshold of 0.6, thereby meeting the criterion required to proceed to the final evaluation phase (T2).

Based on these findings, the final assessment (T2) was conducted on 48 subjects to confirm the stability and reproducibility of the improvements observed after training.

Table 5 (three categories) and Table 6 (five categories) report the agreement values for the final evaluation (T2) of PSIS height measurements obtained separately - first in trunk flexion and second in the standing position (reverse SFT) - by the two examiners in a sample of 48 subjects.

**Table 5:** Intra- and Inter-examiner reliability coefficients for the SFT at T1 (final assessment), using the three-category scale. Abbreviations are as in Table 1.

Comparison 3-category scale	N	Po	Po SE	Po 95% CI (L)	Po 95% CI (U)	Weighted κ (quadratic)	κ SE	κ 95% CI (L)	κ 95% CI (U)	Gwet’s AC2 (quadratic)	AC2 SE	AC2 95% CI (L)	AC2 95% CI (U)	BP-κ (k=3)	BP-κ SE	BP-κ 95% CI (L)	BP-κ 95% CI (U)	PI (0 vs ≠0)	BI (0 vs ≠0)
Intra Examiner 1 — Stand (I vs II)	48	0.938	0.035	0.832	0.979	0.903	0.061	0.76	1	0.903	0.061	0.756	1	0.906	0.052	0.747	0.968	0.354	0.021
Intra Examiner 1 — Flex (I vs II)	48	0.896	0.044	0.778	0.955	0.902	0.046	0.8	0.979	0.902	0.046	0.799	0.979	0.844	0.066	0.667	0.932	0.062	0.021
Intra Examiner 2 — Stand (I vs II)	48	0.979	0.021	0.891	0.996	0.97	0.033	0.882	1	0.97	0.033	0.881	1	0.969	0.031	0.837	0.994	0.312	0.021
Intra Examiner 2 — Flex (I vs II)	48	0.938	0.035	0.832	0.979	0.936	0.039	0.838	1	0.936	0.039	0.838	1	0.906	0.052	0.747	0.968	0.021	0.021
Inter — Stand (Examiner 1 vs Examiner 2, I)	48	0.979	0.021	0.891	0.996	0.97	0.031	0.896	1	0.97	0.031	0.896	1	0.969	0.031	0.837	0.994	0.312	0.021
Inter — Flex (Examiner 1 vs Examiner 2, I)	48	0.917	0.04	0.804	0.967	0.92	0.043	0.819	0.982	0.92	0.043	0.816	0.982	0.875	0.06	0.707	0.951	0.042	0.042
Inter — Stand (Examiner 1 vs Examiner 2, II)	48	0.938	0.035	0.832	0.979	0.903	0.06	0.768	1	0.903	0.06	0.768	1	0.906	0.052	0.747	0.968	0.354	0.021
Inter — Flex (Examiner 1 vs Examiner 2, II)	48	0.917	0.04	0.804	0.967	0.917	0.043	0.814	0.98	0.917	0.043	0.814	0.98	0.875	0.06	0.707	0.951	0	0.042

**Table 6:** Intra- and Inter-examiner reliability coefficients for the SFT at T2 (final assessment), using the five-category scale. Abbreviations are as in Table 1.

Comparison 3-category scale	N	Po	Po SE	Po 95% CI (L)	Po 95% CI (U)	Weighted κ (quadratic)	κ SE	κ 95% CI (L)	κ 95% CI (U)	Gwet’s AC2 (quadratic)	AC2 SE	AC2 95% CI (L)	AC2 95% CI (U)	BP-κ (k=3)	BP-κ SE	BP-κ 95% CI (L)	BP-κ 95% CI (U)	PI (0 vs ≠0)	BI (0 vs ≠0)
Intra Examiner 1 — Stand (I vs II)	48	0.938	0.035	0.832	0.979	0.903	0.061	0.76	1	0.903	0.061	0.756	1	0.906	0.052	0.747	0.968	0.354	0.021
Intra Examiner 1 — Flex (I vs II)	48	0.896	0.044	0.778	0.955	0.902	0.046	0.8	0.979	0.902	0.046	0.799	0.979	0.844	0.066	0.667	0.932	0.062	0.021
Intra Examiner 2 — Stand (I vs II)	48	0.979	0.021	0.891	0.996	0.97	0.033	0.882	1	0.97	0.033	0.881	1	0.969	0.031	0.837	0.994	0.312	0.021
Intra Examiner 2 — Flex (I vs II)	48	0.938	0.035	0.832	0.979	0.936	0.039	0.838	1	0.936	0.039	0.838	1	0.906	0.052	0.747	0.968	0.021	0.021
Inter — Stand (Examiner 1 vs Examiner 2, I)	48	0.979	0.021	0.891	0.996	0.97	0.031	0.896	1	0.97	0.031	0.896	1	0.969	0.031	0.837	0.994	0.312	0.021
Inter — Flex (Examiner 1 vs Examiner 2, I)	48	0.917	0.04	0.804	0.967	0.92	0.043	0.819	0.982	0.92	0.043	0.816	0.982	0.875	0.06	0.707	0.951	0.042	0.042
Inter — Stand (Examiner 1 vs Examiner 2, II)	48	0.938	0.035	0.832	0.979	0.903	0.06	0.768	1	0.903	0.06	0.768	1	0.906	0.052	0.747	0.968	0.354	0.021
Inter — Flex (Examiner 1 vs Examiner 2, II)	48	0917	0.04	0.804	0.967	0.917	0.043	0.814	0.98	0.917	0.043	0.814	0.98	0.875	0.06	0.707	0.951	0	0.042

**Figure 5:**
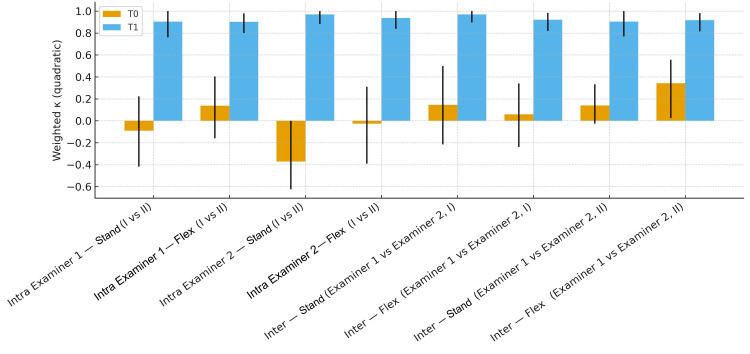
Histograms representing the weighted Cohen’s kappa values. Intra-examiner agreement between the first and second assessments per session, and inter-examiner agreement for the first and second assessments at times T0 and T2, categorized at three levels. Image credit: Saverio Colonna

**Figure 6:**
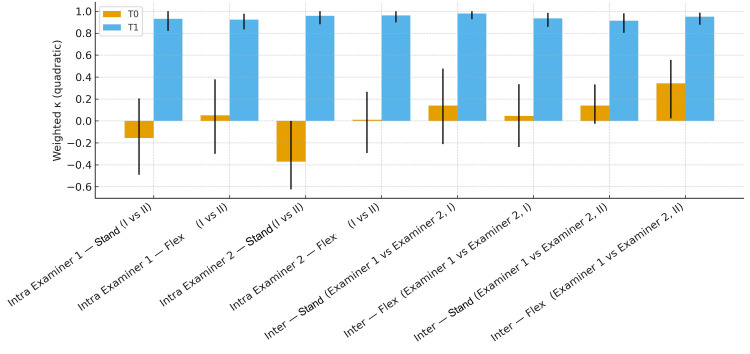
Histograms representing the weighted Cohen’s kappa values. Intra-examiner agreement between the first and second assessments per session, and inter-examiner agreement for the first and second assessments at times T0 and T2, categorized at five levels. Image credit: Saverio Colonna

To better illustrate how the weighted Cohen’s kappa (κw) - the most commonly used statistical index for assessing repeatability in the literature-changed before (T0) and after training (T2), Figure 5 graphically displays the comparison for the three-level categorization and Figure 6 for the five-level categorization.

**Figure 7:**
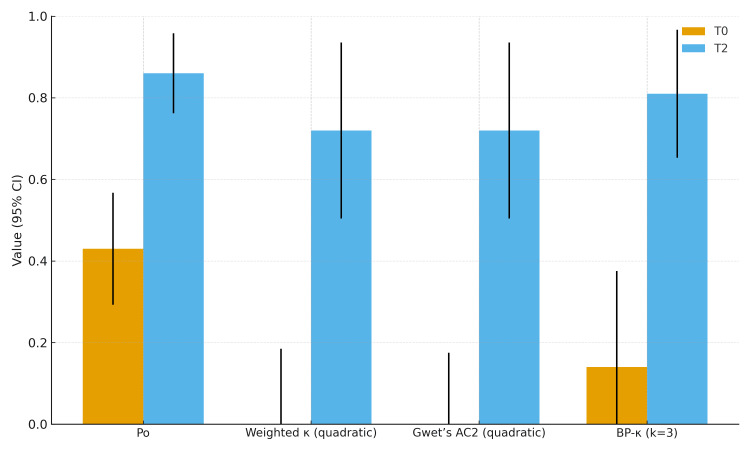
Summary of palpatory reliability at baseline (T0) and after training (T2). Y-axis → Value (95% CI), X-axis → Po (media pesata per N), Cohen’s κ ponderato (quadratico), Gwet’s AC2 (quadratico) e Brennan–Prediger κ (k=3). Image credit: Saverio Colonna

For each metric, the four comparisons (Intra 1, Intra 2, Inter I, Inter II) of T0 and T2 were combined using inverse-variance weighting (1/SE²). Error bars indicate 95% CIs calculated as estimate ±1.96×pooled SE. Input data were extracted from the T0 (n=36) and T2 (n=48) tables. In Figure 7, bars represent pooled estimates (fixed-effect model) with 95% confidence intervals for percent agreement (Po), Cohen’s weighted κ (quadratic), Gwet’s AC2 (quadratic), and Brennan-Prediger κ (k=3).

**Figure 8:**
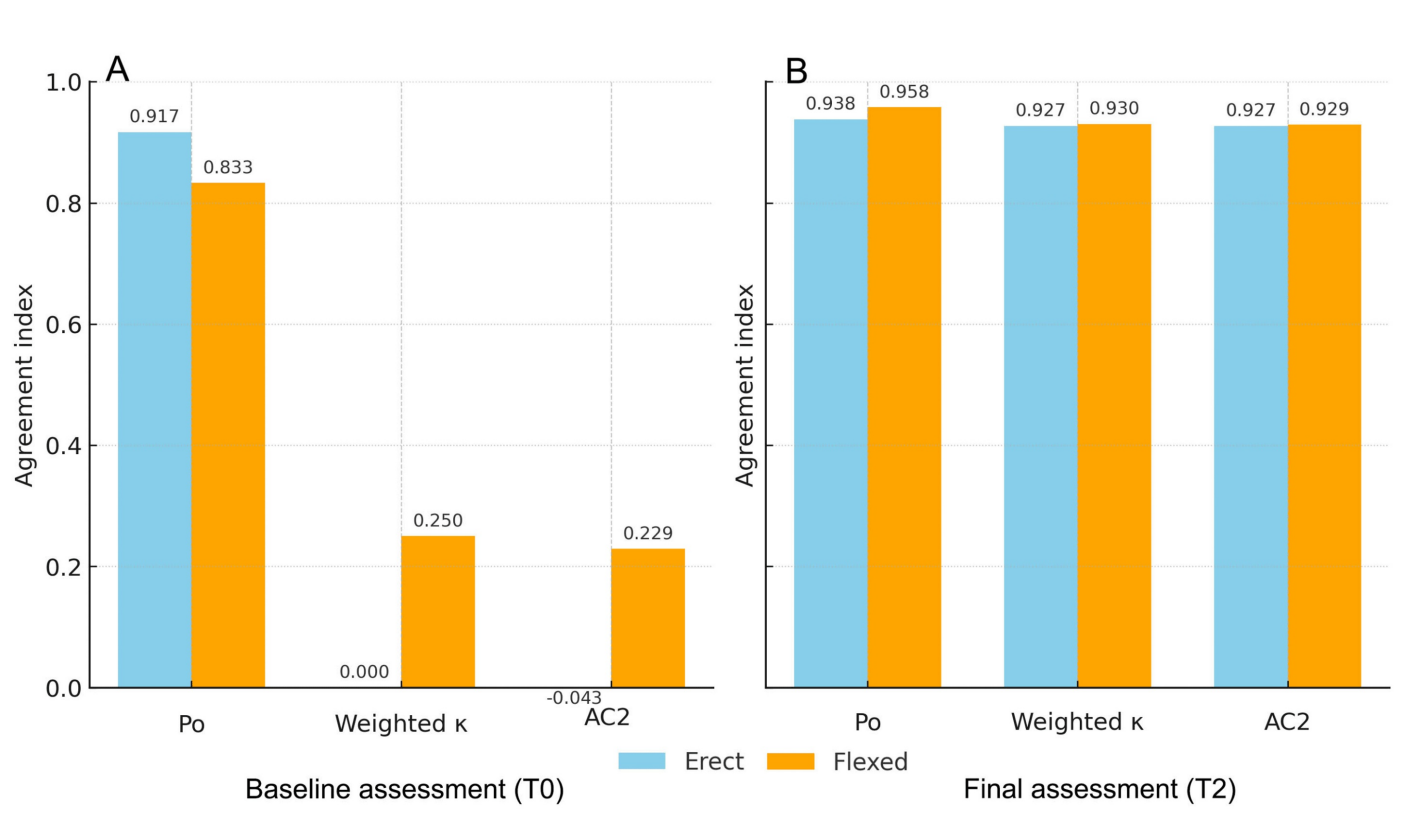
Summary of the reliability of individual assessment moments (flexion and standing) categorized into three levels at times T0 (panel A) and T2 (panel B). Y-axis → Value (95% CI); X-axis → Po (weighted mean by N), weighted Cohen’s κ (quadratic), Gwet’s AC2 (quadratic), and Brennan–Prediger κ (k=3). For each index, the four estimates (Intra1, Intra2, Inter-I, Inter-II) were combined using a fixed-effects model (weight=1/SE²); error bars represent 95% CI=value ±1.96×pooled SE. Input: tables T0 (n=36) and T2 (n=48). Image credit: Saverio Colonna

In the comparison between the two individual assessment phases (flexion and standing), categorized into three levels, the overall agreement obtained at times T0 and T2 was fairly similar; however, the evaluation performed in flexion achieved a slightly higher level of agreement, except for the observed agreement (Po) at time T0 (Figure 8).

Post-Hoc Precision/Power

At T2 (n=48), the achieved precision was consistent with our a-posteriori target (expected 95% CI width ≈0.18-0.24), indicating adequate power (~80%) to discriminate between moderate (κ=0.4) and good (κ=0.6) agreement. At T0 (n=36), the expected precision (95% CI width ≈0.22-0.30) remained acceptable, corresponding to ~70-75% power under the same assumptions.

## Discussion

In the literature, the SFT has been widely questioned, primarily because of its poor reproducibility. Several studies have concluded that both intra- and inter-operator reliability of the SFT do not reach levels sufficient to support its clinical use [45-48].

Some authors have suggested [22,25] that, although the test appears simple, academic training alone is insufficient to ensure reliable performance; rather, specific practical training is required to make it reproducible. Although O’Haire et al. [25] conducted a brief agreement/training session immediately prior to data collection, Fryer et al. [22] were the first to propose a dedicated extracurricular training program - two one-hour sessions - to improve examiner agreement in studies assessing the reproducibility of pelvic palpatory landmarks.

In such training, it is crucial not only to discuss the technical aspects of performing the test but also to define the degree of asymmetry required for examiners to classify a finding as “asymmetric” [22]. Inter-examiner agreement can only be improved by precisely standardizing what constitutes a difference in the symmetry of anatomical landmarks, that is, by determining the specific millimetric deviation considered equal, rather than leaving this evaluation to each examiner’s subjective interpretation.

Visual training for identifying the correct PSIS levels is also of vital importance [37]. It should be emphasized that, although these are defined as palpatory tests, the fingers merely serve to highlight the position of the anatomical landmarks; the final assessment is essentially visual, since only by observing the imaginary line connecting the examiner’s thumbs can one determine whether the PSIS level is horizontal (symmetrical) or oblique (asymmetrical) [37].

The results of this study unequivocally confirm the crucial role of practical training in clinical palpation. Before training, both intra- and inter-operator repeatability were minimal, with values close to zero or even negative (Table 1). After 25 hours of training, agreement improved markedly: for the classical version of the test, evaluating PSIS movement during trunk flexion starting from standing, the weighted kappa values ranged between 0.60 and 0.83 (Table 2).

It should be noted that, in the T2 assessments, the achieved level of agreement in this study may not depend solely on the training performed, but also on the modified PSIS assessment procedure. The reverse SFT introduced in this study begins with the evaluation of PSIS levels while the subject is in trunk flexion and compares these to the levels observed in the standing position. The response criteria remain the same as in the classical test: negative if PSIS movement during standing is symmetrical (within 5 mm), and positive if there is an asymmetrical movement greater than 5 mm, with the positive side corresponding to the PSIS that moves further in the dorsal-caudal direction.

In fact, as described in the literature, palpation of the PSIS - not at the most posteriorly prominent point but slightly inferior to the apex - may cause the fingertips to lose contact with the bony landmark during trunk flexion due to the tensioning of the long dorsal sacroiliac ligament. The hamstring muscles, particularly the biceps femoris [49], have documented fascial connections with the sacrotuberous ligament [49-52]; this ligament, in turn, shows anatomical continuity with the long dorsal ligament, which attaches to the PSIS. Thus, tension in the hamstrings - responsible for controlling anterior pelvic tilt during trunk flexion - increases the tension of the long dorsal ligament [53-55].

This myofascial-ligamentous chain suggests a mechanism of force transmission from the hamstrings to the posterior sacroiliac complex, even though the hamstrings do not have a direct insertion on the PSIS. Because of this tension, during flexion, the examiner may lose contact with the bony landmark and apply either excessive pressure (causing the subject to lose balance) or insufficient pressure (losing tactile contact). This issue is minimized when the PSIS are palpated as the subject returns from flexion to the standing position, because this sequence allows the examiner to apply greater finger pressure to locate and maintain contact with the bony landmark.

The examiners observed that starting the assessment in trunk flexion and then moving to standing - the reverse SFT - improved the precision of PSIS localization and, consequently, examiner agreement. The fourth phase of this work aimed to strengthen both intra- and inter-examiner reliability and to ensure consistent methodology before the subsequent assessments.

Another innovative element introduced in this study was the categorization of PSIS level differences not only into three but also into five categories. This approach allows the test to function not only as a binary orthopedic assessment of sacroiliac pathology, as suggested by some authors [56-58], but also as a functional test that can stratify different degrees of asymmetry.

From a clinical perspective, this functional interpretation is particularly valuable for practitioners who rely on manual evaluation, such as osteopaths, physiotherapists, and other manual therapists. In osteopathic practice, where diagnosis and treatment are grounded in the recognition and correction of somatic dysfunctions, the ability to detect gradations of asymmetry rather than a simple “yes/no” response enhances both diagnostic accuracy and therapeutic precision.

Consequently, the proposed five-level categorization provides clinicians with a more sensitive and informative framework for monitoring functional changes over time and for assessing the effectiveness of manual interventions. For instance, when a manual treatment is performed, it does not necessarily eliminate the dysfunction completely; for example, a subject graded as +1 may not return to 0 immediately after therapy. However, with a five-level categorization, it becomes possible to identify intermediate improvements, such as a change from +2 to +1, which still represents a measurable and clinically meaningful improvement. This provides clinicians with a more sensitive instrument for monitoring progressive functional changes over time and for evaluating the effectiveness of manual interventions beyond a binary outcome.

As a final feature of this study, following a methodology previously reported in the literature [59], the evaluation included not only the definition of test negativity or positivity (right or left) but also the agreement between individual assessments performed with the subject in the standing and flexed positions. Overall, agreement was reported for the position of the PSISs in flexion, in standing, and for the final deduction of the test outcome. When comparing flexion and standing, a slightly higher agreement (both Kw and AC2) was found for the flexion assessment at time T0; this difference decreased at time T2, although values remained higher for flexion (Figure 7). This result is consistent with previous findings reported in the literature [59].

Overall, these findings highlight the importance of structured and shared training programs, combined with methodological refinements such as the reverse SFT and graded scoring systems, to enhance the reliability and clinical usefulness of palpatory assessments in manual medicine.

Limitations of the study

This study has several limitations. First, the healthy volunteer sample limits generalizability to clinical populations, although greater asymmetry in symptomatic patients could facilitate landmark identification [60]. Second, only two examiners were included; adding more raters with diverse experience would better capture inter-examiner variability [33]. Third, the absence of a non-trained control group prevents isolating the specific effect size of the training protocol from potential learning effects. Fourth, the cross-sectional design precludes assessing the temporal stability of training gains. Finally, we evaluated reliability but not validity, as no instrumental or imaging-based reference standard was used.

## Conclusions

The findings of this study indicate that variability and palpatory reliability in osteopathic and manual therapy contexts should not be viewed as intrinsic limitations of the method but rather as consequences of insufficient training and lack of procedural consensus. Incorporating intensive training modules - lasting at least 25 hours - into academic curricula could substantially improve diagnostic accuracy and reduce measurement error. This study provides concrete evidence that palpation, when standardized, can achieve reliability levels comparable to those of other established diagnostic procedures in the health sciences.

Looking ahead, extending the protocol to samples with specific pathologies and applying the training approach in different clinical settings, such as the evaluation of other joint regions, may confirm the broader applicability of this method. Furthermore, future studies should assess not only the reproducibility but also the validity of the SFT by comparing it with an instrumental or imaging-based gold standard, in order to establish whether improved reliability translates into greater diagnostic accuracy and, consequently, clinical usefulness. It is hoped that these findings will encourage further research aimed at consolidating and expanding these results, contributing to the full recognition of palpation as a central tool in clinical assessment.
